# Implementation of multimodal computed tomography in a telestroke network: Five‐year experience

**DOI:** 10.1111/cns.13224

**Published:** 2019-09-30

**Authors:** Carlos Garcia‐Esperon, Frode Soderhjelm Dinkelspiel, Ferdi Miteff, Shyam Gangadharan, Tom Wellings, Bill O´Brien, James Evans, Tom Lillicrap, Jelle Demeestere, Andrew Bivard, Mark Parsons, Chris Levi, Neil James Spratt, for the Northern NSW Telestroke investigators, Rachel Peake, James Hughes, Lisa Dark, Nick Ryan, Matt Shepherd, Osama Ali, James Wills, Fiona Minett, Jaclyn Birnie, Amanda Buzio, Iain Bruce, Alan Tankel, Kim Parrey, Matthew Kinchington, Elizabeth Pepper, Andre Loiselle, Sophie Waller, Alvin Chew, Michelle Russell, Angela Royan, Brett Roworth

**Affiliations:** ^1^ Department of Neurology John Hunter Hospital Hunter New England Local Health District Newcastle NSW Australia; ^2^ Hunter Medical Research Institute and University of Newcastle Newcastle NSW Australia; ^3^ Karolinska Institutet Stockholm Sweden; ^4^ Neurosciences Department Gosford Hospital Central Coast Local Health District Gosford NSW Australia; ^5^ Neurology Department Leuven University Hospital Leuven Belgium; ^6^ Department of Neurology Melbourne Brain Centre at the Royal Melbourne Hospital University of Melbourne Parkville VIC Australia

**Keywords:** acute stroke therapy, core, CT perfusion, multimodal CT, penumbra, telestroke

## Abstract

**Aims:**

Penumbral selection is best‐evidence practice for thrombectomy in the 6‐24 hour window. Moreover, it helps to identify the best responders to thrombolysis. Multimodal computed tomography (mCT) at the primary centre—including noncontrast CT, CT perfusion, and CT angiography—may enhance reperfusion therapy decision‐making. We developed a network with five spoke primary stroke sites and assessed safety, feasibility, and influence of mCT in rural hospitals on decision‐making for thrombolysis.

**Methods:**

Consecutive patients assessed via telemedicine from April 2013 to June 2018. Clinical outcomes were measured, and decision‐making compared using theoretical models for reperfusion therapy applied without mCT guidance. Symptomatic intracranial hemorrhage (sICH) was assessed according to Safe Implementation of Treatments in Stroke Thrombolysis Registry criteria.

**Results:**

A total of 334 patients were assessed, 240 received mCT, 58 were thrombolysed (24.2%). The mean age of thrombolysed patients was 70 years, median baseline National Institutes of Health Stroke Scale was 10 (IQR 7‐18) and 23 (39.7%) had a large vessel occlusion. 1.7% had sICH and 3.5% parenchymal hematoma. Three months poststroke, 55% were independent, compared with 70% in the non‐thrombolysed group.

**Conclusion:**

Implementation of CTP in rural centers was feasible and led to high thrombolysis rates with low rates of sICH.

## 1 INTRODUCTION

Almost 20 years ago, the term telestroke was coined to define the emergent use of telemedicine in acute stroke.^1^ Using a camera and having access to brain computed tomography (CT), neurologists at remote sites were able to determine whether a patient would be candidate for reperfusion treatment with intravenous thrombolysis. It was subsequently shown that telestroke achieved similar safety and outcome results to those in comprehensive stroke centers.^2^, ^3^, ^4^ Importantly, however, acute stroke treatment has changed dramatically over the last few years, endovascular thrombectomy (EVT) is now the standard of care in large vessel occlusion (LVO) strokes. Moreover, recent trials supporting the use of multimodal imaging (including brain noncontrast CT, CT, or MR angiography and perfusion imaging) in the 24‐hour window^5^, ^6^ have driven a need for more sophisticated imaging‐based patient selection. There is also data indicating that such imaging may allow better patient selection and lower rates of symptomatic intracranial hemorrhage (sICH) in those receiving intravenous thrombolysis.^7^, ^8^

John Hunter Hospital is the comprehensive stroke centre for the Hunter and New England regions of New South Wales, Australia. Since April 2013, a telestroke network, where multimodal CT (mCT) was performed routinely at all sites has been developed, aiming to identify potential candidates for thrombolysis and EVT.^9^ Although mCT has typically been restricted to comprehensive stroke centers, recently published trials^5^, ^6^ provided a strong rationale for its use to aid EVT transfer decision‐making from regional and rural centers.

We aimed to a)describe our initial 5‐year experience applying multimodal CT imaging in telestroke and b)determine the influence of mCT on thrombolysis decision‐making. Our principal hypothesis was that the use of mCT implemented in regional hospitals and supported by telestroke would deliver more refined patient selection for thrombolysis—specifically that it would allow selection of those most likely to benefit from therapies—based on presence of a vessel occlusion and “target” mismatch—, and also of those unlikely to benefit, such as stroke mimics, large infarct cores, or small perfusion lesions where the natural history is excellent.^10^

## 2 MATERIALS AND METHODS

### 2.1 Telestroke network

We established a telestroke network to provide acute stroke services to two local health districts (LHD) in New South Wales (Hunter New England and Mid North Coast). The workforce is a combination of stroke neurologists from two different hospitals, John Hunter and Gosford District Hospital. The network commenced in 2013 in Hunter New England, which has a population of 920 370 inhabitants, distributed over an area of 131 785 km^2^ (slightly larger than England, at 130 279 km^2^). Since 2017, the service was extended to the adjoining Mid North Coast Local Health District, with a population of 211 000, and covering 11 335 km^2^.

The network commenced with the first spoke hospital (Manning Base hospital) in April 2013. Since then, four other sites (Tamworth in 2014, Coffs Harbour, Port Macquarie and Armidale hospitals in 2017) have been added. The average distance between the spoke sites and the hub is 284 km (range 167‐386 km). Only one of these hospitals has neurologists or stroke physicians on staff and routinely provided in‐hours thrombolysis after the telestroke service was established.

As part of the network, the local hospitals were equipped with cameras and the physicians were trained in the face arm speech time (FAST) scale. mCT was introduced and performed routinely by trained radiology technicians. Scans were interpreted in the acute phase by the stroke neurologist. For more details, we direct the reader to our published pilot phase experience.^9^

### 2.2 Population

A cohort of patients assessed with telemedicine from April 2013 to June 2018 was collected. The stroke call criteria were occurrence of neurologic symptoms as defined by a positive FAST scale^11^ within 4.5 hours of symptom onset. From November 2017, after publication of the DAWN trial,^5^ the time window for stroke notifications was expanded to 24 hours. Patients dependant on others for personal care prior to the stroke were generally not considered candidates for reperfusion therapies.

### 2.3 Clinical data collection

Clinical data were retrospectively collected from April 2013 to June 2016 and prospectively collected from June 2016 to June 2018. Data collected included baseline demographics, past medical history, NIHSS, multimodal imaging characteristics (vessel status, core and penumbra volumes), acute treatment decision, and clinical outcome. Stroke mimic was defined as a combination of: Atypical stroke presentation, normal follow‐up CT/MRI, and/or clinically determined alternate etiology explaining the event. The modified Rankin Scale (mRS) was used to assess functional outcome after the stroke. Good outcome was defined as a mRS of 0‐2 three months after the stroke.

### 2.4 Imaging protocol and data collection

The mCT imaging protocol included brain noncontrast CT, CT angiography (CTA), and CT perfusion (CTP) at baseline and either NCCT or MRI at 24‐48 hours. The spoke sites used different CT scanners with a‐ to z‐axis coverage between 80 and 150 mm. A 40 mL bolus of iodinated contrast at a rate of 6 mL/s was used to acquire CT perfusion images, lasting between 60 and 72 seconds (depending on the individual scanner protocol). Extracranial CTA was performed afterward, using another 50 mL of contrast agent (rate of 6 mL/s). Intracranial CTA was reconstructed from the CTP acquisition.

All imaging was postprocessed using the commercial software MIStar (Apollo Medical Imaging Technology), which automatically generated cerebral blood volume, cerebral blood flow (CBF), mean transit time and delay time (DT) maps, as well as infarct core and penumbra maps. Penumbra was defined as the tissue with a DT >3 seconds and relative CBF >30% of normal tissue.^12^ Ischemic core was defined as the tissue with a DT >3 seconds and a relative CBF <30% of the contralateral hemisphere.^13^

### 2.5 Thrombolysis decision: multimodal CT versus standard clinical/NCCT criteria

We hypothesized that the use of mCT would allow selection of those most likely to benefit from reperfusion therapies—based on presence of a vessel occlusion and “target” mismatch—, and also of those unlikely to benefit, such as stroke mimics. Our local protocol was that a thrombolysis decision was based on both standard guideline‐based clinical criteria^14^ plus mCT imaging decision assistance using the presence or absence of “salvageable tissue”, defined as at least 15 mL of penumbra assessed by automated perfusion software. The decision for thrombolysis was made by the treating telestroke vascular neurologist in a “real world” clinical practice setting. A patient was considered suitable for thrombolysis using the standard clinical/NCCT criteria if (a) brain NCCT did not show an established ischemic stroke (or bleed), (b) NIHSS score was ≥4, or less if they had significant aphasia or hemianopia, and (c) the patient meets all the other criteria mentioned in the American Stroke Association thrombolysis guidelines.^15^

Presence of sICH was judged using the SITS‐MOST definition, a neurologic worsening of 4 or more points from the baseline NIHSS, or from the lowest NIHSS value between baseline and 24 hours, or leading to death in a patient with Parenchymal Hematoma type 2 (PH2) on follow‐up imaging and was assessed by two experienced independent raters. Clinical diagnosis and all clinical data and imaging results were reviewed and adjudicated by one member of central study team. Stroke mimic was defined as a combination of atypical stroke presentation accompanied by normal 24‐48 hour neuroimaging and a different cause found for the symptoms. Large vessel occlusion was defined as an occlusion in the M1 segment of the middle cerebral artery (MCA), terminal internal carotid artery, basilar occlusion or combined intra‐ and extracranial occlusions (tandem). A vessel occlusion was defined as any visible occlusion identified by the treating stroke neurologist.

### 2.6 Non‐thrombolysed patients

In order to gauge the potential influence of CTP decision‐making on patient outcomes, we assessed outcome not only in those who received thrombolysis, but also in those in whom the use of CTP led to the decision not to thrombolyse and in whom there were no “standard” clinical and NCCT exclusion criteria for thrombolysis. This group differs from the standard clinical/NCCT criteria group mentioned in the above section, since reflecting most current thrombolysis guidelines, we did not apply a NIHSS threshold.

### 2.7 Statistical analysis

Results are presented as mean ± standard deviation (SD) and median [Interquartile range—IQR]. Ethics approval was obtained from the Hunter New England Human Research Ethics Committee (HNEHREC Reference No: 13/02/20/5.06) with a posterior amendment (AU201712‐15).

## 3 RESULTS

**Figure 1:**
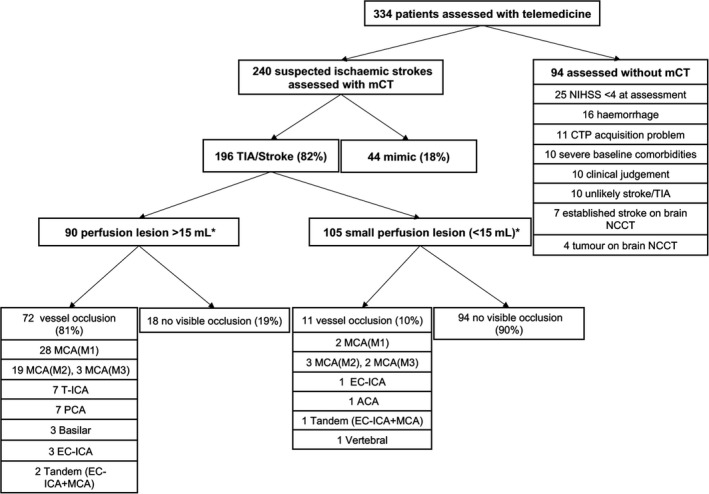
Imaging characteristics of the first 240 patients assessed with multimodal CT. ACA, anterior cerebral artery; EC‐ICA, extracranial internal carotid artery; mCT, multimodal computed tomography; MCA, middle cerebral artery; NCCT, noncontrast CT; and T‐ICA, terminal internal carotid artery. *One missing value

From April 2013 to June 2018, 334 patients were assessed using telemedicine. Of these, 246 (73.6%) had data collected prospectively. mCT was obtained in 240 patients (71.9%). Ninety‐four patients did not receive perfusion imaging. Of these, 27 had contraindications on the brain NCCT (hemorrhage, established stroke or evidence of tumor) and 10 had CTP acquisition‐related issues (artifact, Figure 1).

**Figure 2:**
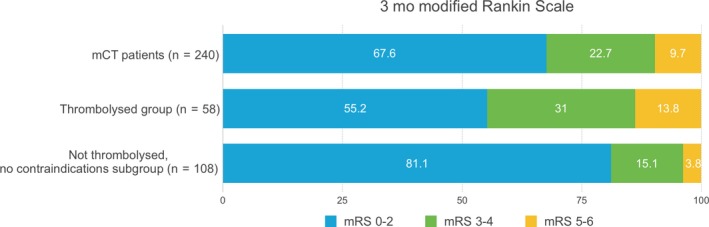
Three months outcome after stroke. mCT, multimodal computed tomography and mRS, modified Rankin Scale. *mCT and non‐thrombolysed groups: 2 missing values in each group

Of the 240 patients assessed with mCT, the mean age was 69 ± 15 years, and 146 (61%) were male. The median baseline NIHSS was 4 [2‐9] (one missing value). An ischemic event was confirmed in 196 patients (175 ischemicstrokesand 21 transientischemicattacks). Forty four patients (18%) were classified as mimics (nine conversion disorders, eight seizures, five migraine with aura, three syncope, two Bell's palsy, two delirium, two vertigo, one drug adverse effect, one sepsis, one tumor, one vocal cord trauma, one hypertensive crisis, and eight nonclassified). Three months after the stroke, 161 of 240 patients (67%) had a good outcome (mRS 0‐2) (2 missing values). 8% of the patients were deceased (Figure 2).

### 3.1 mCT‐based thrombolysis

Of the 240 patients that underwent mCT, thrombolysis was given in 58 (24.2%), 16 were transferred for EVT (7%), and seven received combined therapies (Table 1 and Table S1 of supplementary material). A vessel occlusion was found in 74.1% of the patients; just over half of these being LVO (53.5%), predominantly the MCA‐M1 segment (65% of the LVO group). The median onset to needle time was 172 minutes [140‐220]. The median door to needle time (DTN) was 91 minutes [73‐113], and the median “call to stroke neurologist to needle time” was 65 minutes [IQR 50‐80]. Three months after stroke, 55.2% of the thrombolysed patients were independent, 12.1% had an mRS 3, 20.7% a mRS 4‐5, and 12.1% were dead. Just one patient (1.7%) developed sICH, and two patients had parenchymal hematoma type 1 (3.5%). Data were missing for four patients for alteplase (tPA) administration time and for five patients for time to neurologist call.

**Table 1:** Baseline characteristics and outcome of different groups

Multimodal CT‐assessed patients				
	Whole mCT population (n = 240)	Thrombolysed (n = 58)	tPA eligible per standard clinical/NCCT criteria (n = 80)	Not thrombolysed (n = 108)
Mean age ‐ y (SD)	69 (14.9)	70 (15)	70 (16.7)	67 (15.6)
Baseline mRS 0‐2 – (%)	92	98.3	90	88.9
Median NIHSS [IQR]	4 [2‐9]	10 [7‐18]	8 [5‐15]	2 [1‐4]*
Mean core ‐ mL (SD)	10 (23.9)*	18 (28)	14.3 (24.5)	3 (6.8)
Core > 70 mL – no. (%)	10 (4.1)*	6 (10.3)	6 (7.5)	0
Mean penumbra – mL (SD)	27 (43.4)*	50 (46)	37.3 (45.2)	13 (39.7)
Penumbra < 15 mL ‐ no. (%)	148* (61.7)	17 (29.3)	41 (51.2)	90 (83.3)
Total perfusion lesion <15 mL ‐ no. (%)	140* (58.3)	15 (25.9%)	36 (45)	87 (80.1)
Vessel occlusion – no. (%)	83 (34.6)	43 (74.1)	43 (53.7)	9 (8.3)
Large vessel occlusion and location – no. (%)	43 (17.9)	23 (39.7)	23 (28.7)	
	30 MCA (M1)	15 MCA (M1)	15 MCA (M1)	3 (2.8)
	7 T‐ICA	4 T‐ICA	4 T‐ICA	3 MCA (M1)
	3 tandem	1 tandem	2 tandem	
	3 basilar	1 basilar	1 basilar	
3 months mRS				
0‐2 (%)	67.6**	55.2	57.5*	81.1**
3‐4 (%)	22.7	31	28.8	15.1
5‐6 (%)	9.7	13.8	13.8	3.8

Abbreviations: MCA, middle cerebral artery; mCT, multimodal computed tomography; mRS, modified Rankin Scale;NIHSS, National Institutes of Health Stroke Scale; T‐ICA, terminal internal carotid artery; tPA, tissue plasminogen activator.

* One missing value.

** Two missing values.

### 3.2 Comparison with thrombolysis based on standard clinical/NCCT criteria

Based on standard criteria, 80 patients would have been candidates for thrombolysis; 36 of these were not thrombolysed by the treating clinicians. A visible vessel occlusion was found in 53.7% of the 80 clinically eligible patients, but this was LVO in only 23 (28.7%). Twelve of the clinically eligible but not thrombolysed patients (15%) had no lesion visible on CTP and were subsequently diagnosed as stroke mimics. An additional 36 patients (45%) had a small perfusion lesion (<15 mL). A further 29 patients (36.3%) had penumbra <15 mL and 6 patients had a core >70 mL (7.5%). Moreover, there were 15 patients who would have been ineligible for treatment by standard clinical/NCCT criteria, who had a penumbra >15 mL, two of them received thrombolysis.

### 3.3 Non‐thrombolysed patients

The characteristics of the 240 mCT‐assessed patients and the various subgroups within are shown in Table 1. There were 182 not treated with thrombolysis. Standard contraindications to tPA were present in 74 patients (20 on oral anticoagulation, 20 with extensive hypodensity on brain NCCT, 31 patients outside the tPA time window, 1 postsurgical stroke, 1 previous severe gastro‐intestinal bleed, and 1 had refractory acute hypertension despite treatment. There were 108 patients with no standard clinical contraindications to thrombolysis but who were excluded based on CTP. Just three of these patients had a LVO. One of these was not considered suitable to treatment due to poor baseline function and severe comorbidities, another was not treated with thrombolysis but transferred for clot retrieval and the third patient was asymptomatic at presentation.

Three months after presentation, 86 patients in the nontreated group were independent (81.1%) and 92 (86.8%) were back to their previous level of function. In the subgroup of nontreated patients with a perfusion lesion <15 mL, 79 of the 87 patients (90.1%) were independent or back at their baseline function at 90 days.

## 4 DISCUSSION

We describe our first five‐year experience of a telestroke network with routine use of multimodal CT. Intravenous thrombolysis was delivered to 58 patients, 17.4% of all the calls received during this period. The thrombolysed patients had moderate/severe strokes (median baseline NIHSS of 10), and 74% had a visible vessel occlusion on CTA. Of these patients, 55% were independent three months after stroke. Interestingly, more than 80% of the non‐thrombolysed group were independent 3 months after stroke. A combination of (a) very small or absent perfusion lesions, (b) low percentage of large vessel occlusion (10%), and (c) presence of mimics (44 patients) is the probable explanation for the high rates of good outcome in the non‐thrombolysed group. These data suggest that mCT can identify patients traditionally eligible for thrombolysis, but who actually have an excellent natural history, including stroke mimics and those who are likely already in the process of spontaneous reperfusion (those with significant neurologic deficits but very small cortical perfusion lesions, often in locations not corresponding to their deficits). Our data add to previous observations suggesting that patients with small perfusion deficits may do as well or even better without thrombolysis, calling into question strategies that reward high thrombolysis rates in a relatively undifferentiated population of patients presenting with acute focal neurologic deficit.^7^, ^10^

We compared our thrombolysis outcomes with those from SITS‐MOST registry^16^ and other telestroke networks. The three‐month rate of independence was 55% in SITS‐MOST, identical to this study. SITS‐MOST had a slightly more severe population (baseline NIHSS 12 versus 10 in our population), but lower rate of vessel occlusions. Of the SITS‐MOST patients, 16% were severely impaired after stroke (bedridden/dead), compared with 13.8% in our study. Remarkably, the rate of sICH was 1.7% in our study, compared with 7.3% in SITS‐MOST. This low rate is even more remarkable when one considers that it does not include the many patients who were excluded from thrombolysis based on small or absent perfusion lesions, who would be expected to have very low rates of sICH. A similar very low rate of sICH has also been seen in larger mCT cohorts.^7^, ^17^ Comparing our network with other telestroke programs, our rate of independence of 55% compares favorably to published studies, with rates ranging from 34% to 49%. Those studies reported similar baseline NIHSS (range from 10 to 12, versus 10 in our study).^18^, ^19^, ^20^ All but two previous studies reported sICH rates >5%^18^, ^19^, ^20^, ^21^, ^22^, ^23^; however different sICH definitions were used.

The seniority of the workforce in the emergency departments in our rural catchment is highly variable. Most patients were assessed on site by junior doctors with no prior experience of stroke thrombolysis. Similarly, post‐thrombolysis care in all but one of the participating hospitals was under the management of general physicians, again, most have little experience of post‐thrombolysis care. These outcomes were achieved despite door to needle times that clearly show room for improvement. As it has been the published experience internationally, it takes time to improve door to needle at sites new to thrombolysis. This is the subject of a current practice improvement project across our sites.

We concede that mCT was used to guide treatment decisions where level 1 evidence is lacking. This includes several patients being treated with low NIHSS that would have made them ineligible for treatment per many clinical guidelines. Fifteen patients with penumbra >15 mL (six >50 mL) would not be considered for therapy using traditional clinical criteria, although six of these had a visible vessel occlusion, and two of these were LVOs. However, recent evidence 24, 25 supported the multimodal CT in thrombolysis decision‐making. In this randomized clinical trial and meta‐analysis, multimodal CT was able to identify best responders to thrombolysis in the 4.5‐9 hours window, achieving better functional outcomes, despite a higher rate of symptomatic hemorrhage*.*

A limitation in this observational study is the small part of the data was collected retrospectively (26%), and with relatively small numbers of patients receiving reperfusion therapies, which may affect reliability of estimates of outcomes such as sICH. Nevertheless, our results highlight the feasibility of using mCT in smaller centers lacking stroke neurologists, in particular its potential for reperfusion therapy decision‐making. The data regarding hemorrhage rates were very favorable. Process of care times (acknowledging room for improvement), and outcomes compared favorably with published data, and of note, the outcomes looked even better when based on all those patients who would have been eligible for therapy based on standard clinical and NCCT criteria. In conclusion, our article shows the feasibility of mCT implementation in a rural telestroke network, suggesting enhanced safety of mCT‐based imaging selection.

## APPENDIX 1

Ms Rachel Peake, Dr James Hughes, Dr Lisa Dark, Dr Nick Ryan, Dr Matt Shepherd, Dr Osama Ali, Dr James Wills, Ms Fiona Minett, Ms Jaclyn Birnie, Ms Amanda Buzio, Dr Iain Bruce, Dr Alan Tankel, Ms Kim Parrey, Dr Matthew Kinchington, Dr Elizabeth Pepper, Dr Andre Loiselle, Dr Sophie Waller, Dr Alvin Chew, Ms Michelle Russell, Ms Angela Royan, Mr Brett Roworth
