# Supplementary material for: Implementation of multimodal computed tomography in a telestroke network: Five‐year experience
Source: CNS Neurosci Ther. 2019 Sep 30;26(3):367–73. doi: 10.1111/cns.13224 (PMC7052799; doi:10.1111/cns.13224)
Supplement: Supplementary file 1 [file CNS-26-367-s001.docx]

*Supplementary material*

| *Multimodal CT-assessed patients* | | | | |
| --- | --- | --- | --- | --- |
|  | *Whole mCT population*  *(n=240)* | *Thrombolysed*  *(n=58)* | *tPA eligible per standard clinical/NCCT criteria*  *(n=80)* | *Not thrombolysed*  *(n=108)* |
| *Mean age - yr (SD)* | *69 (14.9)* | *70 (15)* | *70 (16.7)* | *67 (15.6)* |
| *Male sex — no. (%)* | *146 (60.8)* | *37 (63.8)* | *46 (57.5)* | *57 (52.8)* |
| *Hypertension — no. (%)* | *139** (57.9)* | *35 (60.3)* | *47 (58.8)** | *63 (58.3)*** |
| *Hypercholesterolemia — no. (%)* | *60 (25)* | *13 (22.4)* | *18 (22.5)* | *31 (28.7)* |
| *Diabetes mellitus — no. (%)* | *41 (17.1)* | *10 (17.2)* | *14 (17.5)* | *19 (17.6)* |
| *Previous stroke/transient ischemic attack — no. (%)* | *58 (24.2)* | *7 (12.1)* | *18 (22.5)* | *31 (28.7)* |
| *Atrial Fibrillation — no. (%)* | *50 (20.8)* | *15 (25.9)* | *18 (22.5)* | *12 (11.1)* |
| *Ischemic heart disease — no. (%)* | *42 (17.5)* | *10 (17.2)* | *14 (17.5)* | *13 (12)* |

*Table 1.Baseline characteristics of different groups. mCT: multimodal computed tomography, t-PA: tissue plasminogen activator.*

**Three missing values.*

*** Eleven missing values.*
